# The S100 Protein Family as Players and Therapeutic Targets in Pulmonary Diseases

**DOI:** 10.1155/2021/5488591

**Published:** 2021-06-18

**Authors:** Zeeshan Sattar, Alnardo Lora, Bakr Jundi, Christopher Railwah, Patrick Geraghty

**Affiliations:** ^1^Division of Pulmonary and Critical Care Medicine, Department of Medicine, State University of New York Downstate Health Sciences University, 450 Clarkson Avenue, Brooklyn, NY 11203, USA; ^2^Department of Cell Biology, State University of New York Downstate Health Sciences University, 450 Clarkson Avenue, Brooklyn, NY 11203, USA

## Abstract

The S100 protein family consists of over 20 members in humans that are involved in many intracellular and extracellular processes, including proliferation, differentiation, apoptosis, Ca_2_^+^ homeostasis, energy metabolism, inflammation, tissue repair, and migration/invasion. Although there are structural similarities between each member, they are not functionally interchangeable. The S100 proteins function both as intracellular Ca^2+^ sensors and as extracellular factors. Dysregulated responses of multiple members of the S100 family are observed in several diseases, including the lungs (asthma, chronic obstructive pulmonary disease, idiopathic pulmonary fibrosis, cystic fibrosis, pulmonary hypertension, and lung cancer). To this degree, extensive research was undertaken to identify their roles in pulmonary disease pathogenesis and the identification of inhibitors for several S100 family members that have progressed to clinical trials in patients for nonpulmonary conditions. This review outlines the potential role of each S100 protein in pulmonary diseases, details the possible mechanisms observed in diseases, and outlines potential therapeutic strategies for treatment.

## 1. Introduction to S100 Proteins

In multiple diseases, numerous exogenous and endogenous factors trigger inflammatory cascades. A family of endogenous proinflammatory mediators named damage-associated molecular patterns (DAMPs; also known as alarmins) have become of special interest in the past few decades. During stress reactions or cell injury, DAMPs are released and activate inflammatory pathways [1]. DAMPs function independently when they are intracellular and act in a cytokine-like manner when they are extracellular. Of note, it is suggested that the term “DAMPs” is too broad of a term to characterize all endogenous molecules linked to an inflammatory pathway; thus, there is an ongoing debate on defining DAMPs and classifying them [2]. One family of DAMPs that have been identified to play a significant role in the host immune response in multiple diseases is the S100 protein family.

S100 proteins are involved in both intracellular and extracellular processes including cell apoptosis, migration, protein phosphorylation, calcium balance, differentiation, proliferation, and inflammation [3–6]. The term S100 was first used by Blake Moore to describe proteins that were soluble in 100% saturated ammonium sulfate in 1965 [7]. There are now 25 such S100 proteins/complexes described, which include 16 S100A proteins (S100A1-S100A16) as well as others (such as S100B, S100G, S100P, and S100Z) [8]. The S100 protein family is exclusively expressed in vertebrates. It consists of small (10-14 kDa), acidic, and calcium-binding proteins (CaBPs) with two distinct EF-hand motifs (helix-loop-helix) [9]. This subfamily of CaBPs exists mainly as homodimers but can also exist as monomers (only S100G is stable in this configuration), heterodimers (S100A1/S100B and S100A8/S100A9), or multimers intra- and extracellularly. Their expression is tissue and cell-type specific [10]. Along with Ca^2+^, S100 proteins also bind many other transition metal ions (e.g., Fe^2+^, Cu^2+^, Mn^2+^, Zn^2+^, and Ni^2+^) that result in a conformational change allowing interaction with target proteins [11, 12]. S100 proteins act as DAMP molecules and can work as stimulatory ligands for both immune and nonimmune cells, such as endothelial cells [13]. They do so by binding pattern recognition receptors (PRRs) such as toll-like receptor 4 (TLR4) as well as non-PRR DAMP receptors such as advanced glycation end products (RAGE) [14]. By binding to these receptors, S100 proteins trigger downstream nuclear factor-*κ*B (NF-*κ*B), which results in upregulation of the proinflammatory gene expression [15, 16]. In this review, we outline the potential role of the S100 protein members in pulmonary diseases and detail potential molecular mechanisms they may play in disease initiation and progression. Equally, we outline potential therapeutic approaches to treat pulmonary diseases by targeting S100 proteins.

## 2. S100 Proteins: Activation, Expression, Interacting Proteins, and Targets

S100 proteins interact with a variety of target proteins including enzymes, cytoskeletal subunits, receptors, transcription factors, and nucleic acids. Therefore, through structural changes, interactions with Ca^2+^, posttranslational modifications, receptor-mediated transduction, and direct responses S100 proteins mediate many processes.

### 2.1. S100 Protein Structure, Calcium Bind, and Posttranslation Modifications

Pending on the cellular and extracellular location of S100 proteins, they can interact with many proteins and form dimers. There are two critical steps for S100 protein activation: Ca^2+^ binding [17] and dimer formation [18]. Typically, the S100 proteins are observed as symmetric dimers, with each S100 subunit containing four *α*-helices [4]. Several heterodimers are reported among family members *in vitro*, but only the S100A8-S100A9 heterodimer is believed to play a significant role *in vivo* [19]. S100 subunits contain two Ca^2+^-binding domains, a carboxy-terminal canonical domain composed of 12 amino acids and an amino-terminal domain composed of 14 amino acids [4]. Both are connected by a 10-12 residues region, crucial for target interactions. Ca^2+^-binding affinity drastically increases when a target engages [20–22]. Upon binding Ca^2+^, S100 proteins undergo conformational rearrangement exposing a hydrophobic cleft that is required for target binding. Therefore, S100 target binding is mostly Ca^2+^-dependent. Individual S100 family members exhibit differences in surface geometries, hydrophobic residue distribution, and charge density [23]. Several S100 proteins undergo posttranslational modifications, such as oxidative modification, s-nitrosylation, phosphorylation, covalent modification, transglutaminase 2-mediated crosslinking, sumoylation, S-glutathionylation, cysteinylation, the formation of intra- and intersulfinamide bonds, or intracellular localization [24–29]. The activity of S100 proteins is regulated by metal ions, such as calcium, zinc, and copper, that modulate the folding and oligomerization of the protein [30]. Regarding pulmonary disease, few studies focus on the structure or modification of S100 proteins but merely their presence or the impact of deficiency or inhibition on disease initiation or progression.

### 2.2. S100 Expression Profile

Each S100 family protein is encoded by a separate gene, mostly but not limited to chromosome 1q21 [9]. When examining the expression of each S100 gene between different cell types or tissues, despite structural similarities and clustered genes, each S100 gene has a very specific expression pattern [31–33]. Therefore, we must not expect similar expression patterns for each S100 gene member. Dysregulated expression of multiple S100 family members occurs in most diseases. Epigenetic events are reported in various medulloblastoma cell lines resulting in dysregulated S100 gene expression [34]. DNA de-methylation and hypomethylation [35] and micro-RNA regulation are reported in the regulation of the S100 gene expression. CpG islands are observed in the 5′ regulatory regions of S100A2, S100A6, S100A10, and S100A11 genes, within the proximal promoter and the first two introns [36]. Methylation of these islands is typically associated with a repressed chromatin state and transcription inhibition. But not all S100 genes contain these CpG islands, such as S100P and S100Z genes. Equally, several S100 genes are reported to be upregulated due to several extracellular factors, such as oxidative stress, certain cytokines, and growth factors in many types of cells [37]. Within pulmonary diseases, the expression of S100 genes is reported to be primarily triggered by extracellular responses. Importantly, different subsets of monocytes can have different expression profiles of S100 proteins [38]. Our group demonstrated that the protein tyrosine phosphatase (PTP1B) could negatively regulate the S100A9 expression and reduced S100A9 stimuli responses via regulation of TLR4 signaling [39]. However, there are several other means to regulate the S100A9 expression as it is sensitive to the Src kinase inhibitor PP2 [40] and the STAT3 expression [41]. Therefore, the S100 gene expression varies depending on the cell type, tissue, external stimuli, age, and possible sex.

### 2.3. Secretion of S100 Proteins

Little is known about the mechanisms that mediate the secretion of S100 proteins. The S100 proteins lack a leader sequence and are not secreted via the classical Golgi pathway. The secretion of S100 proteins appears to occur either passively upon cell necrosis or actively following cell activation. Several S100 proteins undergo secretion following stimulation by particular cell activators, such as serotonin-receptor agonists [42], antidepressants, glutamate, adenosine, IL-1*β* [43], lysophosphatidic acid, and changes in extracellular Ca^2+^ and K^+^ levels [44]. Some S100 proteins have affinities to lipid structures that may allow them to translocation across the plasma membrane following cell stress or activation [45]. Equally, S100 proteins can interact with phospholipid-binding proteins, such as S100A10 binding to Annexin A2 [46], to induce exocytosis of intracellular S100 proteins to the extracellular domain. S100 protein members, S100A8/S100A9, may be released from necrotic myeloid cells or actively secreted following translocation to the membrane, utilizing an intact microtubule network and PKC activation [47]. Oxidative stress can induce the release of S100 proteins from several cells. S100A8/S100A9 could be released neutrophil extracellular traps (NETs). Therefore, S100 proteins may be readily secreted within the lungs. Expression levels and plasma or BALF levels of several S100 proteins are observed to be altered in the lungs of several pulmonary diseases, as outlined in Section 3 and Table 1.

### 2.4. S100 Protein Receptors and Targets

**Figure 1:**
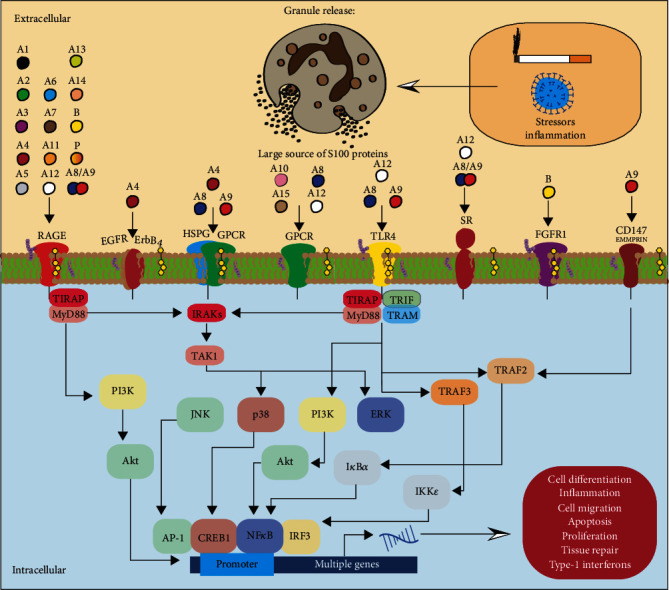
Extracellular functions of S100 proteins. Several factors can trigger S100 proteins to be released or secreted from multiple cell types, including granulocytes. The extracellular S100 proteins interact with several pattern recognition receptors, resulting in proinflammatory signaling pathways that promote cell differentiation, inflammation, migration, apoptosis, proliferation, tissue repair, and a robust type-1 interferon response. Only a portion of the RAGE, HSPG/GPCR, TLR4, and CD147 responses are shown here. RAGE: receptor for advanced glycosylation end products; EGFR: epidermal growth factor receptor; ErbB: v-erb-b2 avian erythroblastic leukemia viral oncogene homolog; HSPG: heparan sulfate proteoglycans; GPCR: G*α*9-coupled receptor; TLR4: toll-like receptor 4; SR: scavenger receptor; FGFR1: fibroblast growth factor receptor 1; CD147: cluster of differentiation 147; EMMPRIN: extracellular matrix metalloproteinase inducer; TIRAP: TIR domain containing adaptor protein; MyD88: myeloid differentiation factor 88; IRAK: interleukin 1 receptor-associated kinase; TAK1: transforming growth factor-*β*- (TGF*β*-) activated kinase 1; TRIF: TIR-domain-containing adaptor protein inducing IFN*β*; TRAM: translocating chain-associated membrane; TRAF: TNF receptor-associated factor; PI3K: phosphoinositide 3-kinases; Akt: protein kinase B; JNK: JUN N-terminal kinase; ERK: extracellular signal-regulated kinase; AP-1: activator protein 1; CREB1: cAMP-responsive element-binding protein 1; NF-*κ*B: nuclear factor-*κ*B; IRF: interferon regulatory factor; I*κ*B*α*: nuclear factor of kappa light polypeptide gene enhancer in B cell inhibitor, alpha; IKK: inhibitor of NF-*κ*B kinase.

Once released to the extracellular space, S100 proteins trigger immune cell activation through binding to different cell surface receptors. S100 proteins primarily trigger inflammation responses through their interactions with toll-like receptor (TLR) 4 and receptor for advanced glycation end products (RAGE) [48]. S100A8 and A9 are known to bind to TLR4 [48], while S100A7, S100A12, S100A8/A9, and S100B interact with RAGE [16, 49, 50]. By interacting with RAGE and/or TLR4, S100 proteins can activate mitogen-activated protein kinases (MAPK) responses and transcription factors, such as NF-*κ*B, resulting in the production of proinflammatory cytokines [50, 51]. S100A6 activates RAGE to promote apoptosis [52]. However, S100B inactivates RAGE [53]. S100 proteins are also known to interact with extracellular matrix metalloproteinase inducer (EMMPRIN) (also known as CD147), G-protein-coupled receptor (GPCR), CD36 [54], FGFR1 [55], CD166 antigen [56], IL-10 receptor [57], neuroplastin-*β* [58], CD68 [59], and ErbB4 [60] (see Figure 1). It is important to note that S100 protein heterodimers and the presence of calcium may also influence different receptor responses.

## 3. Linking S100 Proteins to Pulmonary Disease Outcomes

Multiple sources suggest that S100 protein family members augment the inflammatory response and disease outcomes in a variety of different pulmonary diseases.

### 3.1. S100A1

S100A1 is a small (~10 kDa) protein that predominantly functions to modulate the Ca^2+^ milieu, energy metabolism, and contraction of the cardiac myocytes [61]. It is also shown to be present in vascular endothelial cells and smooth muscle cells, and its absence is associated with a hypertensive phenotype in animal models partly secondary to endothelial cell dysfunction [62, 63]. Teichert-Kuliszewska et al. demonstrated that S100A1 is also expressed in the lung vascular endothelium, and its deficiency in knockout (KO) mice leads to increased right ventricular (RV) systolic pressure (RVSP) as well as aberrant endothelial-dependent relaxation in response to acetylcholine and decreased availability of nitric oxide (NO) thus predisposing to pulmonary hypertension [61]. Pigs embolized with Sephadex developed RV hypertrophy and, interestingly, showed increased RV S100A1 expression and mild pulmonary hypertension (pH) [64].

### 3.2. S100A2

S100A2 is linked to both suppressions of tumor progression as well as being a promoter of carcinogenesis [65–69]. Its role in non-small-cell lung cancer (NSCLC) was elucidated by Feng et al. who suggested that hypermethylation of the promoter region of the S100A2 gene led to its downregulation in the early stages of NSCLC carcinogenesis thus contributing to tumor progression [68]. In contrast, some data suggest that overexpression of S100A2 may indicate a poor prognosis in stage 1 NSCLC [70]. EGF-stimulated EGFR phosphorylation induces the S100A2 expression, and S100A2 exhibits antitumor activity by reducing the rate of tumor growth when overexpressed in nude mice with NCI-H2172 cell tumor xenograft model [71].

### 3.3. S100A3/S100A13

Mutation of the S100A3 gene leads to the replacement of arginine with cysteine at the 77th position of the S100A3 protein along with a mutation of the S100A13 gene leading to decreased expression of both proteins. This is implicated in an atypical genetic variant of early onset interstitial pulmonary fibrosis [72]. The decreased expression of these proteins leads to abnormal calcium homeostasis intracellularly, decreased tolerability of oxidative stress, and altered expression of extracellular matrix proteins. In addition to its role in idiopathic pulmonary fibrosis (IPF), S100A3 is also a potential drug target in lung cancers. Gianni et al. showed that knockdown of S100A3 leads to degradation of RAR*α*, a transcription factor that plays a role in the antiproliferative effects of All-trans retinoic acid (ATRA) [73], by proteasomes thus inducing resistance to the antiproliferative and differentiating effects of ATRA on lung cancer cells [74]. Tumor microvascular density (MVD) is an indicator of tumor neovascularization which advocates recurrence, potential for metastases, and survival of cancerous cells. Overexpression of S100A13 in stage I NSCLC has been associated with enhanced intratumoral MVD. This suggests that S100A13 plays a role in lung cancer progression and metastasis [75]. Similarly, it is associated with a more aggressive invasive phenotype of lung cancer [76].

### 3.4. S100A4

S100A4 was originally labeled as a specific marker of fibroblasts in different tissues and was termed as FSP-1 (fibroblast-specific-protein-1), but further studies showed that S100A4 is also involved in the fibrogenic of mesenchymal progenitor cells in the lung thus contributing to the pathogenesis of IPF [77]. S100A4 is released by M2 alveolar macrophages, which then activates fibroblasts [77]. Inhibition of the S100A4 expression with niclosamide improved survival in a bleomycin-induced fibrosis model in mice [77]. Bleomycin significantly increases the S100A4 expression in alveolar macrophages [77]. Extracellular S100A4 activates lung fibroblasts by the upregulation of *α*-SMA, type I collagen, and sphingosine-1-phosphate (S1P) [78]. S100A4 is also involved in the pathogenesis of lung cancer through inhibition of autophagy [79], a process by which defective proteins and organelles are degraded in lysosomes to maintain the cellular milieu [80, 81]. S100A4 inhibits starvation-induced autophagy, thus promoting the proliferation of NSCLC cells through activation of the Wnt/*β*-catenin pathway in a RAGE-dependent manner [79]. S100A4 regulates oxygen consumption rates, mitochondrial activity, ATP production, and glycolytic activity by regulating mitochondrial complex I subunit NADH dehydrogenase (ubiquinone) Fe-S protein 2 (NDUFS2) [82]. Interestingly, S100A4 has some antitumorigenesis properties as 10% of *S100a4*^−/−^ mice develop spontaneous tumors that are p54 positive, including bronchioalveolar carcinomas [83]. This loss of *S100a4* in mice coincided with elevated *S100a3* and *S100a5* expression in several tissues [83].

### 3.5. S100A5/S100G

The prognostic value of high levels of expression of S100A5 and S100G mRNA is associated with worse overall survival in the nonsmoking NSCLC patients [84]. Further studies are warranted to further elucidate the role of S100A5 as a prognostic marker in lung cancer. However, differential expression of S100A5 is observed in primary human bronchial epithelial (HBE) isolated from healthy, asthmatic, and COPD donors grown at the air-liquid interface when exposed to fine particulate matter [85].

### 3.6. S100A6

S100A6 plays a role in the proliferation, apoptosis, cytoskeleton modulation, and stress-induced responses of normal adult cell types as well as several tumorous cells [86]. It is overexpressed in lung cancer cells and serum from cancer patients [87, 88]. S100A6 plays a role in the proliferation, invasion, migration, and angiogenesis of lung cancer cells by degrading p53 acetylation, a process by which p53 gets activated and performs its role as a tumor suppressor [89]. The S100A6 expression is downregulated by overexpression of the microRNA, miRNA-193a, thereby increasing p53 acetylation [90]. Hematopoietic stem cells from *S100a6*^−/−^ mice have impaired self-renewal and regenerative capacity [91]. Chronic exposure to cigarette smoke increases S100A6 lung levels in Wistar rats [92]. Mice deficient for the cystic fibrosis (CF) transmembrane conductance regulator (CFTR) gene develop multiple complications, including severe pulmonary disease. Male *Cftr*^−/−^ mice have elevated lung *S100a8* and *S100a9*, while demonstrating reduced *S100a6* and *S100a13* [93]. A recent study suggests that there is an S100A6 profile in systemic sclerosis and may represent a potential biomarker [94]. Finally, although the levels of S100A6 along with S100A11 are different between IPF, sarcoidosis, and COPD subjects and healthy controls, there was no prognostically significant [95].

### 3.7. S100A7

The majority of early work looking at S100A7, also called psoriasin, focused on psoriasis [96, 97]. However, several studies suggest that S100A7 may have a role in several respiratory diseases, including lung cancer [98], asthma [99], rhinosinusitis [100], pulmonary complications of systemic sclerosis [101], and bacterial exacerbations [102]. S100A7 is a chemotactic protein for CD4+ T cells and neutrophils present in the skin epidermis [103]. It also functions as a direct bactericidal protein [104]. Kim et al. demonstrated that levels of S100A7 and S100A8/A9 were reduced in chronic rhinosinusitis (CRS) patients with a mixed ball of bacteria and fungi compared to a fungal ball [100]. This implies that the barrier function of the nasal epithelial cells is reduced in mixed fungal and bacterial infections thus putting patients at risk of developing an invasive fungal infection.

S100A7 also appears to play a role in the pathogenesis of NSCLC. A study investigating the TGF*β* signaling pathway in the progression of NSCLC demonstrated that the TGF*β*-induced long noncoding RNA (TBILA) binds to S100A7 thus activating the S100A7/JAB1 pathway leading to NSCLC proliferation and metastasis [98]. The S100A7 expression is induced by activation of the Hippo pathway and acts as a facilitator in the adenosquamous transition of lung cancer cells [105]. S100A7 levels are higher in lung squamous carcinoma and have the potential to be used as a diagnostic marker as its levels are elevated in cancerous cells as compared to nonneoplastic cells [106]. Knockdown of S100A7 leads to decreased phosphorylation of NF-кB thus inhibiting tumor proliferation. This marks S100A7 as a potential therapeutic target for the treatment of lung squamous cell cancers [106].

In asthma, S100A7 plays a role in the interplay between the proinflammatory cytokine IFN-*γ* and IL-22. The expression of S100A7 is induced by IL-22, and these effects are in turn inhibited by IFN-*γ* [99]. The expression of S100A7 is enhanced in cases where *S. aureus* is found in the respiratory tract thus stressing the role of this molecule in the epithelial barrier of the lower respiratory tract [102]. S100A7 levels correlate with lung involvement in systemic sclerosis, and the increased expression of psoriasin in the whole saliva has a specificity of 50% and a sensitivity of 85% in detecting lung involvement in this condition [101].

### 3.8. S100A8 and S100A9

As mentioned above, S100A8 and S100A9 proteins are upregulated in *Cftr*^−/−^ animals, thus suggesting a role of these proteins in the disease process of CF [93]. The anti-inflammatory role of S100A8 in emphysema was investigated by Lin et al., and low intracellular S100A8 levels were observed in emphysema that correlated with disease severity. Furthermore, this protein also has a cytoprotective role, as it is shown that the knockout of this protein leads to oxidative stress-induced cell apoptosis while elevated levels prevent cellular injury [107]. Interestingly, the S100A8/A9 heterodimer may play a role in acute exacerbations of COPD, as it is negatively associated with FEV1% in these patients [108]. Similarly, another study observed elevated S100A8 and S100A9 in COPD samples [109]. Our group has identified S100A9 signaling in smoke and age-related COPD progression [110]. Levels of S100A9 are elevated in older animals as well as animals exposed to cigarette smoke [110]. The unique intracellular and extracellular responses of both of these proteins require further investigation in the lungs. Similar to COPD, it appears that S100A8, S100A9, and the S100A8/A9 heterodimer have opposite effects in acute lung injury (ALI). S100A8 similarly inhibits ALI to dexamethasone [111] while S100A9 and calprotectin (the S100A8/A9 complex) promote neutrophil influx by increasing mast cell degranulation and upregulation of particular chemokines. S100A8 is IL-10 dependent while S100A9 and calprotectin do not induce IL-10 in the airways or tracheal epithelial cells [112].

The S100A8/A9 complex is expressed in tuberculosis (TB) [113–115], as S100A8/A9-expressing neutrophils are observed to assemble in granulomas and S100A8/A9 levels correlated with active disease [113]. This protein also mediates neutrophil accumulation in chronic TB by upregulating the integrin molecule CD11b [116]. The levels of both of these proteins in BALF and sera of asthmatic patients correlate with elevated levels of serum IgE [117]. In addition, they inhibit the apoptosis of neutrophils by increasing the levels of MCP-1, IL-6, and IL-8 through the PI3K/AKT/MAPK/NF-*κ*B pathway [118]. S100A9 enhances the migration of fibrocytes in asthma exacerbations as well as chronic obstructive asthma [119]. S100A9 and/or S100A8/A9 may also have an anti-inflammatory function in asthma by downregulating the function of CD4^+^ Treg cells [120].

Saito et al. elucidated the role of alarmins in chronic lung allograft dysfunction (CLAD), a major contributor to morbidity and mortality in long-term survivors of lung transplantation [121]. The two subtypes of CLAD, restrictive allograft syndrome (RAS) and bronchiolitis obliterans syndrome (BOS), exhibited distinct expression patterns of alarmins including S100A8, S100A9, S100A8/A9, S100A12, S100P, HMGB1 (high mobility group box-1), and soluble RAGE suggesting different biologic profiles of CLAD subtypes. S100A8/A9 overexpression could be involved in the interaction of metastatic lung cancer cells and bone marrow adipocytes leading to bone destruction [122]. S100A8 and S100A9 are upregulated in the acute exacerbations of idiopathic pulmonary fibrosis (IPF) suggesting roles of different signaling pathways like Clathrin-mediated endocytosis signaling, atherosclerosis signaling, and IL2 signaling in the pathogenesis of acute exacerbations of IPF [123]. Serum S100A8/A9 levels are significantly increased in patients with IPF compared with healthy controls and correlate with the diffusing capacity for carbon monoxide (DLCO) and the composite physiologic index [124]. Finally, elevated levels of S100A9 protein are present in the nasal tissues of chronic rhinosinusitis (CRS) subjects. S100A9 mediates the MMP3 expression, resulting in nasal epithelial cell proliferation [125].

### 3.9. S100A10

S100A10 is believed to be a signature gene involved in blood pressure regulation and maybe a potential target for the detection of risk, prevention, and treatment of hypertension [126]. S100A10 is also one of the cytoskeletal regulatory proteins. It enhances endothelial cell barrier function and is recruited to the caveolin-induced microdomains by high-molecular-weight hyaluronan [127] and potentially could contribute to syndromes of endothelial dysfunction like acute lung injury and sepsis. In primary lung cancers, S100A10 overexpression is associated with higher stage and invasiveness of lung adenocarcinoma [128] as well as lymphatic invasion, cancer stage progression, and poor prognosis in lung squamous cell carcinoma [129]. Increased expression of Kallikrein-related peptidase 6, a serine protease that is normally expressed in mammary tissue, is linked with the enhanced metastatic potential of breast cancer to the lungs and elevated expressions of S100 proteins, like S100A4 and S100A11 [130]. On the contrary, expression at physiological levels leads to suppression of some S100 proteins (S100A4, S100A10, S100A13, S100A16) and thus inhibition of lung metastases.

### 3.10. S100A11

S100A11 also called S100C or calgizzarin, overexpression is linked with alterations in K-RAS mutated lung adenocarcinomas as well as poorly differentiated lung adenocarcinomas [131]. It is associated with shorter disease-free survival in these tumors. S100A11 levels are increased in the plasma of patients with pulmonary arterial hypertension (PAH) [132]. Hypoxia-induced mitogenic factor, a protein upregulated in animal models of PAH and asthma, is associated with S100A11-mediated smooth muscle cell migration, vesicular exocytosis, and nuclear activation [133].

### 3.11. S100A12

S100A12 is strongly expressed in the early stages of ALI and early acute respiratory distress syndrome (ARDS) [134]. Possible effects of overexpression include activation of pulmonary endothelium, leukocyte extravasation, and neutrophil accumulation leading to lung injury. Thus, this protein has the potential to be used as a marker of inflammation in ARDS. Contrary to ARDS and ALI, one study demonstrated that this protein blunts inflammatory responses in asthma in murine models [135]. It is also reported to be elevated in COVID-19 patients [136, 137].

### 3.12. S100A14

The S100A14 expression is associated with a subset of lung adenocarcinoma and has a strong correlation with the invasive and migratory nature of lung adenocarcinoma cells [138]. Its expression is also increased in NSCLC where it has been demonstrated to act as a target of miR-335-3p contributing to NSCLC progression through the pathway involving cancer susceptibility candidate-9, a long noncoding RNA (lncRNA) [139].

### 3.13. S100A15 and S100A16

Both S100A15 (*S100A7A* gene) and A16 expression are altered in lung cancers. Increased S100A15 expression and decreased DNA methylation of its gene promoter region are associated with poor outcomes in lung adenocarcinoma cases and potentially greater metastasis [140]. The S100A16 expression is increased in NSCLC [141] as well as adenocarcinoma [142] and is a potential marker for prognosis in patients with these varieties of lung cancer.

### 3.14. S100B

S100B is a nervous system-specific protein and is expressed in glial and Schwann cells [143]. Its levels can rise in response to increased blood-brain barrier permeability [143, 144]. Increased expression of this protein is associated with brain metastases in patients with small cell lung cancer (SCLC) and may indicate poor prognosis as higher levels are linked with shorter survival time [145]. In contrast, increased S100B mRNA expression is associated with better survival in smoking NSCLC patients [84].

### 3.15. S100P

The S100P expression is associated with the migration, invasiveness, and metastasis of lung cancer [146] as well as the migration of NSCLC [147]. S100P, as an immune-associated gene (IAG) signature, can potentially be used in risk score models to predict the overall survival, stage, lymph node accumulation, tumor metastasis, B cells, and dendritic cell infiltration of lung adenocarcinoma [148]. S100P is sequestered by NORAD, an lncRNA, thereby playing a role in the inhibition of metastatic potential of lung and breast cancers [146]. The transcriptional activation of S100P can be regulated by Porcupine proteins thus contributing to the development of NSCLC [149]. S100P may also play a role in the pathogenesis of pulmonary hypertension in patients with systemic sclerosis as it is overexpressed in these patients [150].

## 4. Role and Mechanisms of S100 Proteins in Pulmonary Diseases

### 4.1. S100 Protein-Mediated Signaling in Pulmonary Diseases

In noncancerous pulmonary diseases, several S100 proteins (S100A1, A4, A8, A9, A12, and B) are noted to induce multiple pathways associated with pulmonary disease phenotypes. Here, we will outline the known signaling transduction of these S100 proteins in noncancerous pulmonary diseases. The primary pathways investigated are TLR4 and RAGE-mediated (Figure 1 and Table 1).

Deficiency of *S100a1* results in pulmonary hypertension in mice due to enhanced eNOS activity and nitric oxide levels, via Akt/ERK1/2 pathways, and reduced endothelial cell survival [61]. Alternatively, overexpressing S100A4 in mice results in the development of RAGE-mediated pulmonary arterial hypertension in females [151]. Stimulation with 17*β*-estradiol increases the S100A4 expression and proliferation in human pulmonary artery smooth muscle cells, and this proliferation is prevented by blocking RAGE signaling [151]. S100A4 also prevents autophagy and induces proliferation in A549 and Lewis lung carcinoma cells, via the RAGE and Wnt pathways [79].

Similarly, S100A9 promotes proliferation of lung fibroblasts and upregulated expression of proinflammatory cytokines (IL-6, IL-8, IL-1*β*) and collagen type III, via ERK and NF-*κ*B signaling [152]. The S100A8/S100A9 dimer induces the secretion of cytokines, such as MCP-1, IL-6, and IL-8, from bronchial epithelial cells in a TLR4/Akt mediated pathway [118]. This process prevented neutrophil apoptosis in cell coculture experiments [118]. Cigarette smoke is known to modulate the signaling of TLR4 [153], RAGE [154], and EMMPRIN [155]. Our group determined that enhanced S100A9 signaling coincides with lung damage during respiratory syncytial virus (RSV) infection in mice [39]. We determined that S100A9 is enhanced by cigarette smoke exposure and further enhanced during viral exacerbations in mice and human primary lung cells [39]. We also recently demonstrated that cigarette smoke-induced S100A9 contributes to loss of lung function, airspace enlargements, elastin degradation, enhanced phosphorylation of ERK and c-RAF, and altered expression of MMP-3, MMP-9, MCP-1, IL-6, and KC/IL-8 [110]. Equally, elevated S100A9 levels in the lungs correlate with aging [110]. This is also reported in the central nervous system [156] and may occur with other S100 proteins within the lung as S100B blood levels are age-related [157, 158]. Interestingly, p-PKA*α* interacts with S100A8 in lung tissue obtained from smokers [107]. This interaction alters the phosphorylation of serine within S100A8 and subsequent S100A8 protein destabilization and degradation [107]. Loss of S100A8 increases the percentage of apoptotic A549 cancer cells [107]. S100A8/S100A9 also acts as a chemotactic factor by inducing neutrophil adhesion [159] and can induce ROS-mediated apoptosis, autophagy, mitochondrial damage, and lysosomal activation in various cell types, including lymphocytes, macrophages, endothelial cells, and tumor cells [51]. Expressions of *S100a8* and *S100a9* are both elevated in the lungs of CF transmembrane conductance regulator (*Cftr*) knockout mice, while *S100a6* and *S100a13* are reduced [93]. S100A8/A9 also regulates CD11b expression and neutrophil recruitment during chronic tuberculosis [116]. Interestingly, the downregulation of S100A8 and S100A9 is associated with the differentiation of myeloid cells toward dendritic cells and macrophages [160]. Alternatively, *S100A9*^−/−^ mice displayed significantly enhanced allergic airway inflammation upon exposure to *Alternaria alternata*, including IL-13, CCL11, CCL24, serum IgE, lung eosinophils, IL-13^+^IL-5^+^CD4^+^ T-helper type 2 cells, airway resistance, and elastance [120]. Recently, we observed that S100A9 expression in chronic rhinosinusitis samples coincides with elevated plasma proteases, and S100A9 protein enhances MMP-7 and MMP-3 production and proliferation in the CCL-30 cell line [125].

Finally, S100A12 and S100B signaling is observed in the lungs. Mice expressing the human S100A12 gene have reduced peribronchial and perivascular inflammation, mucus production, eosinophilia, and airway responsiveness [135]. This was associated with S100A12 induced *Fas* expression and activation of caspase 3 in cultured airway smooth muscle cells, thereby leading to reduced airway smooth muscle [135]. In bronchial epithelial cells, the S100B expression is upregulated due to MyD88-dependent activation of canonical NF-*κ*B in the early stages of fungal infection but later becomes downregulated via TLR-3/9-dependent signaling [161]. CD8+ T cells and NK cells also express and secrete S100B following stimulation [162].

### 4.2. S100 Proteins in Tissue Repair

Despite S100 proteins being associated with cellular and tissue damage, several members (including S100A7, S100A8/A9, S100A12, and S100A15) are reported to also play a critical role in tissue repair [163]. These S100 proteins are reported to be involved in tissue repair in atherosclerosis, dermatitis, and arthritis [14]. Importantly, S100A4 promotes muscle tissue repair to maintain contractility following heart injury [164]. Within pulmonary diseases, little is reported on the role of S100 proteins in tissue repair. However, intracellular S100A8 was recently shown to protect type II pneumonocytes from smoke-induced cell death [107]. Hiroshima and colleagues demonstrate that S100A9 and S100A8/A9 can reduce neutrophil influx in the LPS model of acute lung injury [112]. Since S100A8/A9 and LPS target TLR4, S100A8/A9 may compete for TLR4 binding with LPS. Interestingly, *S100a9*^−/−^ mice accumulated a lower frequency of CD4+ T regulatory (Treg) cells following exposure to *Alternaria alternata* [120], leading to more lung damage. Equally, despite S100B responses activating intracellular TLRs, during a pulmonary *Aspergillus fumigatus* infection, S100B can itself resolve inflammation via transcriptional inhibition of itself [161]. Therefore, several members of the S100 family could influence cell fate, inflammation, and tissue remodeling within the lungs. There may also be several feedback loops that initially trigger inflammation and subsequently regulates the resolution of these responses to minimize damage and facilitate repair.

## 5. Targeting S100 Proteins to Treat Pulmonary Diseases

Due to the involvement of S100 proteins in the pathogenesis of numerous diseases as highlighted in this review and mouse models suggest that genetic deletion has minimal effects on normal physiology, there is increasing interest in therapeutic targeting these proteins. Clinical trials to date have primarily focused on nonpulmonary diseases such as systemic lupus erythematosus (SLE), ischemic heart failure, and rheumatoid arthritis. We will discuss S100 inhibitors and clinical trials undertaken with these therapeutics to date (summarized in Table 2).

### 5.1. Clinical Trials to Date Utilizing the Quinoline-3-Carboxamide Derivatives S100 Protein Inhibitors

In the past decade, several small molecules were identified to block the hydrophobic cleft required for the recognition of S100 targets and thus block their activity, such as paquinimod (ABR-215757), tasquinimod (ABR-215050), and laquinimod (ABR-215062). They are quinoline-3-carboxamide derivatives that primarily block the interaction of S100A8 and S100A9 with RAGE and TLR4 [165, 166]. Laquinimod, a derivative of linomide (Roquinimex), interferes with S100 and its receptor RAGE binding and is proposed as a treatment for multiple sclerosis [167]. Here, we will briefly outline several of the clinical outcomes utilizing these quinoline-3-carboxamide derivatives S100 protein inhibitors primarily in prostate cancer, SLE, and multiple sclerosis. However, few studies in human clinical trials report their findings as of the time of this paper, and pulmonary outcomes are limited.

Phase II randomized, double-blind, placebo-controlled study in men with minimally symptomatic metastatic castrate-resistant prostate cancer (CRPC), tasquinimod improves progression-free survival in patients with metastatic castration-resistant, possibly by reducing the recruitment of MDSCs and inhibiting metastasis [168, 169]. However, when tasquinimod was studied in a global phase III randomized trial in men with bone CRPC and while it significantly improved radiographic progression-free survival, this did not result in an overall survival benefit [170]. A recent phase II study failed to demonstrate the clinical activity of tasquinimod in heavily pretreated patients with advanced hepatocellular, ovarian, renal cell, and gastric cancer, and many experienced adverse events such as fatigue, nausea, decreased appetite, and vomiting [171].

There are multiple published findings from clinical trials with laquinimod, most notably in multiple sclerosis populations. In a large, multicenter phase III clinical trial (ALLEGRO), laquinimod was well tolerated [172]. The most common adverse events were elevated liver enzymes (3.6% on laquinimod, 0.4% on placebo), abdominal pain, back pain, and cough. However, another trial with laquinimod (BRAVO [173]) in patients with relapsing multiple sclerosis showed conflicting results with the efficacy of laquinimod in reducing relapses and MRI measures of inflammation of multiple sclerosis patients. Recently, laquinimod did not reach the primary endpoint of reduction in confirmed disability progression in a phase 3 trial of patients with relapsing multiple sclerosis [167].

Paquinimod is reported to be beneficial in several animal models including systemic sclerosis [174], COPD [110], type 1 diabetes [175], and SLE [176]. Recently, our group observed that treatment of cigarette exposed mice with paquinimod, an S100A9 inhibitor, could preserve lung function [110]. In an SLE-prone mouse study where the animals developed significant glomerulonephritis resulting in hematuria and proteinuria, treatment with paquinimod was comparable to prednisolone or mycophenolate treatment [176]. A phase 1b study in humans demonstrates good tolerance of paquinimod at 3 mg/kg, but several adverse events, such as arthralgia and myalgia, were reported with the highest dose levels of paquinimod (4.5 mg/day and 6.0 mg/day) [176]. However, although a phase 2 study was initiated, no results have been released as of the time of this paper [177].

### 5.2. Other Potential Therapeutic Intervention of S100 Proteins

Here, we will briefly outline some alternative approaches to target S100 proteins that could be applied to lung diseases. These include gene delivery approaches, inhibitory antibodies and peptides, vaccines, and chemical inhibitors (see Table 2). Several new compounds were recently identified specifically for S100 proteins that inhibit the interactions of S100P [178], S100A4 [179, 180], S100A9 [179], S100A10 [181], and S100B [182, 183] with their targets. The antihistaminic drug, cromolyn, binds to S100A1, S10012, S100A13, and S100P and disrupts S100P interacting with RAGE [178]. A cromolyn analog which inhibits S100P was used in pancreatic cell line and mice models to reduce tumor growth and metastasis [178]. Another anti-inflammatory antiallergic immunomodulatory drug, Amlexanox, interacts with S100A1, S100A4, and S100A13 and alters S100 protein signal transduction [184]. Alternatively, phenothiazines interact with multiple S100 family members, such as S100A4 [22], and may influence their signaling.

Equally several compounds are known to inhibit the S100A4 expression, with calcimycin (a calcium ionophore), niclosamide (an antihelminth drug), and sulindac (a nonsteroidal anti-inflammatory drug) are all reported as inhibitors of S100A4 transcription [185, 186]. Interestingly, calcimycin can augment surfactant secretion in cultured type II airway epithelial cells (AEC II) [187, 188]. Equally, niclosamide increases the sensitivity of cancer cells for radiation therapy in lung cancer [189] and alleviates pulmonary fibrosis *in vitro* and *in vivo* by attenuation of epithelial-to-mesenchymal transition, matrix proteins, and Wnt/*β*-catenin signaling [190]. Sulindac is also linked to lung cancer therapy and pulmonary fibrosis [191]. Whether these responses are due to S100A4 are unknown. Pentamidine is deemed an S100B inhibitor as it reduces several inflammatory markers such as MDA, PGE2, and IL-1, which in turn downregulated S100B [192]. By blocking S100B activity, pentamidine rescues expression of the tumor suppressor factor wtp53 and restores proapoptotic responses in colon cancer [193]. Alternatively, other groups have tried to reduce S100B levels, using arundic acid, and found that treatment with arundic acid at 24-48 hours after the induction of ischemia significantly decreased infarct volumes by approximately 40% [194].

Other approaches to modulate S100 protein activity include neutralizing antibodies [39, 195, 196] and peptide-Fc fusion proteins (peptibodies) directed against S100A8 and S100A9 [197]. Antibody-based therapies are an interesting approach for several of the S100 proteins as they possibly could reduce toxicity and off-target effects but may be limited to only extracellular S100 proteins. Treatment with an anti-S100A4 antibody decreased signs of allergy in a mouse model as well as in allergen-challenged T cells from allergic patients [198]. Inhibiting extracellular S100 protein signaling may be beneficial for COPD, as intracellular S100A8 protects against type II pneumocyte cytotoxicity [107], while extracellular S100A9 contributes to disease progression [110]. Some inhibitory peptides can penetrate cells with S100B inhibitory peptides reported to penetrate tumor cells and reduce growth in a melanoma xenograft model [199]. Immunization with an anti-S100A9 vaccine in a mouse ischemic stroke inhibited long-term thrombus formation, through inhibition of increased S100A9/CD36 signaling, without risk of bleeding or adverse autoimmune responses [200]. In rodent [201] and pig [202] cardiac disease models, adenoviral-associated vector S100A1 gene delivery to cardiac tissue normalized low S100A1 levels and restore cardiomyocytes physiologic contractility, restored cardiac performance, and left ventricular remodeling. Therefore, many approaches exist to modulate S100 protein signaling but most require testing in the setting of pulmonary diseases.

## 6. The Influence of Neutrophils in the Release of S100 Proteins in Pulmonary Diseases

Finally, we want to briefly discuss the importance of S100 proteins and neutrophils in pulmonary diseases. Neutrophils are the first responders to the site of inflammation and are essential for microbial containment, eradication, and host survival. Dysregulated neutrophil responses are central to the pathophysiology of many inflammatory lung diseases. Approximately 45% of the cytosolic proteins in neutrophils are constituted with S100A8, S100A9, and S100A12 and are released upon injury or infection [203, 204]. Following activation of neutrophils, the S100 proteins are secreted or released and function in an autocrine and paracrine manner. The released S100 proteins induce the production of proinflammatory cytokines, neutrophil degranulation and chemotaxis, leukocyte adhesion and endothelial transmigration, and increased effects of lipopolysaccharide on phagocytes and cells (Figure 1). At the site of inflammation, calprotectin acts as a chemotactic factor by inducing neutrophils adhesion [159] and thereby further exaggerates neutrophil responses.

Rammes et al. have suggested that in monocytes, the nonclassical S100A8/A9 secretion constitutes an intact microtubule network and PKC activation [114]. However, in neutrophils, the process by which S100A8/A9 is secreted is not fully elucidated and may be different from the pathway in monocytes since the S100A8/A9 complex is detected in NETs. Secretion of S100A8/A9 is dependent on the production of ROS and required K^+^ exchanges through the ATP-sensitive K^+^ channel [205]. Schenten et al. indicated that S100A9 is phosphorylated at threonine 113 by the MAPK p38 in activated neutrophils and released to the extracellular space by the process of NETosis [27, 206, 207]. Equally, S100A9 is an activator of the *β*-2 integrin Mac-1 (CD11b/CD18) on neutrophils [208] and could influence neutrophil trafficking, phagocytosis, ROS production, and T cell activation [209]. However, further research is needed to elucidate the signaling pathway.

With the new human viral disease severe acute respiratory syndrome coronavirus 2 (SARS-CoV-2), there is emerging data identifying the roles of S100 proteins to coronavirus disease 2019 (COVID-19). In preclinical models of SARS-CoV-2 infected animals and patients, there are increased levels of immature neutrophils and dramatically upregulated S100A8 levels [210]. In addition, multivariable analysis of patients' samples demonstrated that elevated S100A9 was independently associated with mortality [211]. Serum S100A8/A9 levels in COVID-19 patients are linked to severity and in-hospital mortality [212] and early indicator of respiratory failure [210]. This suggests that S100 proteins of neutrophil origin could significantly contribute to inflammation and respiratory outcomes in COVID-19 subjects. Higher blood frequency of NETs and neutrophil activation markers including S100A8/A9 are observed in COVID-19 cases associated with thrombosis [213] and in COVID-19 with severe pulmonary outcomes [214]. The presence of S100A8/A9 in fecal is linked to intestinal inflammation in COVID-19 patients [215, 216]. Equally, monocytes with low expression of HLA-DR and high expression of S100A8, A9, and 12 are strongly associated with severe COVID-19 [137]. Serum S100B COVID-19 patients significantly correlate with disease severity and are associated with inflammation markers (ferritin, C-reactive protein, procalcitonin) and organ damage markers (alanine aminotransferase, creatinine) [217]. Recently, paquinimod was shown to resolve SARS-CoV-2 mediated pneumonia by reducing the viral load and a subset of neutrophils in mice [218]. Therefore, neutrophil-associated S100 proteins may represent key players in the pathogenesis of many pulmonary diseases, including COVID-19.

## 7. Conclusions

In the past two decades, our knowledge of the roles of S100 proteins in pulmonary diseases has grown, and many novel approaches were identified to target members of this protein family as therapeutic options. Despite considerable progress in S100 protein biology, we still know little about posttranslational modifications or heterodimer formation impact on S100 signaling, and the COVID-19 pandemic highlights the potential role of S100 proteins in tissue damage.

## Figures and Tables

**Table 1:** S100 proteins involved in pulmonary diseases.

Protein	Receptor and interacting proteins	Major cells expressing	Pulmonary disease	Study	
S100A1	RAGE	Macrophages, dendritic, epithelial, and endothelial cells	Reduced in pulmonary hypertension	[61]
S100A2	RAGE	Cancer cells, basal, club and ciliated cells	Reported both induced and reduced in non-small-cell lung carcinoma	[71]
S100A3	RAGE, retinoid receptor, RAR*α*, and PML-RAR*α*	Endothelial, epithelial, and lymphatic cells	Reduced in both pulmonary fibrosis and lung cancer models	[72] [74]
S100A4	TLR4, IL-10R, EGFR, ErbB4, HSPG, GPCR, and RAGE	Tumor cells, T cells, neutrophils, and macrophages	Increased in both lung NSCLC and pulmonary fibrosis	[77] [79]
S100A5	RAGE	Macrophages and cancer cells	Increased in nonsmoking NSCLC	[84]
S100A6	RAGE	Most lung cells but predominantly neutrophils, macrophages, and NK cells	Increased in lung cancer cells, idiopathic pulmonary fibrosis, sarcoidosis, and COPD Reduced in CF	[90] [95] [95] [95] [94]
S100A7	RAGE	Epithelial cells and neutrophils	Reduced in rhinosinusitis Increased in lung NSCLC, squamous cell cancer, asthma, bacterial-associated exacerbations, and pulmonary involvement in systemic sclerosis	[100] [98, 105, 219, 220] [99] [102] [101]
S100A8	TLR4, heparan sulfate and N-glycans, S100A9, and RAGE	Many tissues but predominantly monocytes, granulocytes, and epithelial cells	Increased in cystic fibrosis, extracellular levels in COPD, chronic tuberculosis, asthma, acute lung injury, restrictive allograft syndrome, and COVID-19 Reduced intracellular levels in COPD	[93] [107, 109] [116] [118] [112] [121] [136, 137]
S100A9	TLR4, heparan sulfate and N-glycans, RAGE, S100A8, and EMMPRIN	Many cell types but predominantly macrophages, granulocytes, and epithelial cells	Increased in COPD with and without AE, cystic fibrosis, lung cancer, chronic tuberculosis, IPF, acute, exacerbations, asthma, acute lung injury, restrictive allograft syndrome, rhinosinusitis, and COVID-19	[39, 108–110] [93] [122, 221] [116] [123, 124] [118–120] [112, 125] [121] [125] [136, 137]
S100A10	GPCRs, serotonin receptors, CCR10, and AnxA2	Many cell types but predominantly macrophages, granulocytes, and epithelial cells	Increased in lung adenocarcinoma, hypertension, and acute lung injury	[129, 130] [126] [127]
S100A11	RAGE	Ubiquitous expression in various tissues and cell types	Increased in lung adenocarcinoma, hypertension, idiopathic pulmonary fibrosis, sarcoidosis, and COPD	[131, 222] [223] [95] [95] [95]
S100A12	TLR4, RAGE, N-glycans, scavenger receptors, and GPCR	Granulocytes and monocytes	Increased in asthma, COPD, ARDS, restrictive allograft syndrome, and COVID-19	[134, 135] [109] [224] [121] [136, 137]
S100A13	RAGE	Ubiquitous expression in various tissues and cell types	Increased in pulmonary fibrosis and lung cancer cells	[72] [75, 225]
S100A14/S100A11P	RAGE	Epithelial cells and cancer cells	Increased in lung adenocarcinoma and NSCLC	[139, 226, 227]
S100A15	GPCR	Cancer cells and neutrophils	Increased in lung adenocarcinoma	[140]
S100A16	Unknown receptor interaction but binds to S100A14	Cancer, epithelial, endothelial cells, and fibroblasts	Increased in lung adenocarcinoma and NSCLC	[141, 142]
S100B	RAGE and FGFR1	Cancer cells, dendritic cells, and lymphocytes	Increased in lung SCL but reduced in NSCLC	[145] [84]
S100G	Annexin A10	Epithelial and cancer cells	Increased in lung NSCLC	[84]
S100P	RAGE and p53	Epithelial and cancer cells	Increased in restrictive allograft syndrome, lung NSCLS, adenocarcinoma, and pulmonary arterial hypertension	[121] [146, 148, 149] [150]
S100Z	S100A1, S100A3, and S100B	Monocytes and dendritic cells	No known pulmonary link	

**Table 2:** Current drug development targeting S100 proteins.

Therapeutic approach	Agent	Possible S100 protein interaction	Current status	Study
Small molecule inhibition of S100 proteins Quinoline-3-carboxamide derivatives	Tasquinimod (ABR-215050)	Oral administration that blocks S100A8 and S100A9 interacting with RAGE and TLR4	Phase II randomized, double-blind, placebo-controlled studies in men with minimally symptomatic metastatic CRPC	[168–170]
	Paquinimod (ABR-215757)	Oral administration that blocks S100A8/S100A9 interacting with TLR4	Phase I study demonstrated good tolerance in SLE patients, while a phase II study data never published	[176, 177]
	Laquinimod (ABR-215062)	Oral administration that blocks S100A8 and S100A9 interacting with RAGE and TLR4	Conflicting data in several multicenter phase II and III clinical trials in multiple sclerosis populations	[167, 172, 173]
Inhibitors of S100 proteins	Cromolyn (cromoglicic acid)	An antihistaminic drug binds to S100A1, S10012, S100A13, and S100P and disrupts interactions with RAGE	FDA approved 20 years ago as an antihistaminic drug, as a nasal spray (NasalCrom)	[178]
	Amlexanox	Interacts with S100A1, S100A4, and S100A13 to prevent their signaling	Used to treat recurrent aphthous ulcers but discontinued in the USA In Japan, it is used to treat bronchial asthma, allergic rhinitis, and conjunctivitis	[184] [228]
	Phenothiazines, such as trifluoperazine	Disrupt the interaction of S100A4 with myosin-IIA	Used to treat psychotic disorders, anxiety, nausea, and vomiting caused by chemotherapy	[229]
Indirect inhibitors of S100 protein signaling	Pentamidine	Downregulates inflammation mediated S100B	An antimicrobial agent to treat African trypanosomiasis, leishmaniasis, Balamuthia infections, babesiosis, and Pneumocystis pneumonia	[192]
	Arundic acid	Reduces S100B levels	A multicenter, dose-escalating, randomized, double-blind phase I trial was performed in acute ischemic stroke	[194, 230]
	Calcimycin (A23187)	Inhibit S100A4 expression	A calcium ionophore used against gram-positive bacteria and fungi. Also, in *in vitro* fertilization and to make artificial liposomes for cancer drugs	[185]
	Niclosamide (niclocide)	Inhibit S100A4 expression	Oral administered antihelminth drug	[231]
	Sulindac	Inhibit S100A4 expression	A nonsteroidal anti-inflammatory drug	[186]
Gene delivery approaches	Adenoviral-associated vector S100A1 gene delivery	Restored S100A1 levels to restore cardiomyocytes physiologic contractility, cardiac performance, and left ventricular remodeling	Only tested in animal models	[201, 202]
S100 neutralizing antibodies	Anti-S100A4, anti-S100P, anti-S100A9	Prevent extracellular signaling of S100 proteins	Only tested in animal models	[39, 195, 196]
S100 neutralizing peptides	Peptide-Fc fusion proteins (peptibody)	Depletes myeloid-derived suppressor cells getting to tumor and releasing S100 proteins	Only tested in animal models but reduced tumor growth	[197, 199]
Vaccines targeting S100 proteins	Anti-S100A9 vaccine	Prevent S100A9/CD36 signaling in a mouse ischemic stroke model	Only tested in animal models	[200]
